# Supplementary material for: IMPA2 Downregulation Enhances mTORC1 Activity and Restrains Autophagy Initiation in Metastatic Clear Cell Renal Cell Carcinoma
Source: J Clin Med. 2020 Mar 30;9(4):956. doi: 10.3390/jcm9040956 (PMC7230261; doi:10.3390/jcm9040956)
Supplement: Supplementary file 1 [file jcm-09-00956-s001.pdf]

Supplementary Table S1. Cox univariable and multivariable analyses under the condition of overall survival probability in association with *IMPA2/mTORC1* geneset mRNA expression levels and pathological stage derived TCGA cohort with clear-cell renal cell carcinoma.

**Overall Survival (*n* = 493)**				
**Variables**	**Univariable (HR, 95%CI)**	***P***	**Multivariable (HR, 95%CI)**	***P***
Age				
<60	1	NA	1	NA
≥60	1.76 (1.29–2.40)	<0.001	1.66 (1.21–2.28)	0.002
gender				
female	1	NA	1	NA
male	0.94 (0.69–1.28)	0.690	0.81 (0.58–1.12)	0.207
pT				
T1-T2	1	NA	1	NA
T3-T4	3.02 (2.22–4.11)	<0.001	0.51 (0.43–1.53)	0.516
pM				
M0	1	NA	1	NA
M1	4.27 (3.12–5.83)	<0.001	2.28 (1.54–3.38)	<0.001
stage				
I-II	1	NA	1	NA
III-IV	3.62 (2.64–4.99)	<0.001	2.31 (1.12–4.76)	0.023
grade				
I-II	1	NA	1	NA
III-IV	2.57 (1.83–3.61)	<0.001	1.50 (1.03–2.18)	0.034
IMPA2 levels				
High	1	NA	1	NA
Low	2.02 (1.48–2.77)	<0.001	1.54 (1.09–2.16)	0.014
mTORC1 levels				
Low	1	NA	1	NA
High	1.83 (1.34–2.49)	<0.001	1.33 (0.96–1.85)	0.085
IMPA2/mTROC1 levels				
High/Low	1	NA	1	NA
Low/High	3.06 (2.00–4.68)	<0.001	1.10 (0.79–1.52)	0.578

HR and CI denote hazard ratio and confident interval, respectively.

Supplementary Table S2. The relationship between *IMPA2*/mTORC1 geneset levels and the clinicopathological characteristics of TCGA cohort with clear cell renal cell carcinoma.

**Clinicopathological Characteristics**	***n***	***IMPA2*/mTORC1 Geneset Levels, *n* (%)**			***P*** [a]
		**High/Low (*n* = 143)**	**Others (*n* = 208)**	**Low/High (*n* = 142)**	
Age					
<60	244	72(29.5%)	105(43.0%)	67(27.5%)	
≥60	249	71(28.5%)	103(41.4%)	75(30.1%)	0.808
gender					
female	168	70(41.7%)	64(38.1%)	34(20.2%)	
male	325	73(22.5%)	144(44.3%)	108(33.2%)	<0.001
pT					
T1-T2	308	109(35.4%)	133(43.2%)	66(21.4%)	
T3-T4	185	34(18.4%)	75(40.5%)	76(41.1%)	<0.001
pM					
M0	415	132(31.8%)	178(42.9%)	105(25.3%)	
M1	78	11(14.1%)	30(38.5%)	37(47.4%)	<0.001
stage					
I-II	291	105(36.1%)	123(42.3%)	63(21.6%)	
III-IV	202	38(18.8%)	85(42.1%)	79(39.1%)	<0.001
grade					
G1-G2	224	86(38.4%)	100(44.6%)	38(17.0%)	
G3-G4	269	57(21.2%)	108(40.1%)	104(38.7%)	<0.001

[a] *P* values were derived with a two-sided Pearson chi-square test.
